# Cointegration analysis of vulnerability index and standardised precipitation index in Mafeteng district, Lesotho

**DOI:** 10.4102/jamba.v9i1.330

**Published:** 2017-11-24

**Authors:** Bernard M. Hlalele

**Affiliations:** 1 School of Open Learning, University of the Free State, South Africa

## Abstract

Given the high poverty levels in Africa, with most countries’ economy and populations’ livelihood dependent on rain-fed agriculture, land degradation among other environmental hazards has proven to be a major threat to economic growth and food insecurity, respectively. Drought, which is on the increase at the global level and said to create over 78% of other hazards, has aggravated land degradation. Dry conditions lessen soil particles cohesion force, thereby increasing susceptibility of such soils to be lost by wind and water. The current study aimed at estimating land degradation from drought hazard index, standardised precipitation index (SPI) over the drought declared district of Mafeteng Lesotho. Data were provided by Lesotho Meteorological Services for a period of 30 years (1984–2014). All missing values that existed in the collected precipitation data were filled with average values of the months with data. The computation of SPI was performed by using DrinC software in SPI-3 and SPI-Annual time step. The results revealed a constant condition of land degradation vulnerability over a 30-year period, implying a continuous loss of soil fertility, agricultural gross domestic product (GDP), water and bio-energy, malnutrition and increased poverty levels.

## Introduction

Land is one of the most vital natural capital assets; it is therefore crucial to address its challenges at the nexus of poverty, food insecurity, water and bio-energy, particularly with reference to the rural poor and most vulnerable people (Tesfahunegn, Tamene & Vlek 2014). At a global level, land degradation has resulted in an average of about 5% loss of agricultural GDP which is actually twice as high in the developing countries (United Nations Convention to Combat Desertification [UNCCD] 2013: The European Commission Joint Research Centre 2015). Moreover, land use and vegetation cover changes have a significant influence on the carbon fluxes and green gas emissions that subsequently alter the atmospheric composition and radiative force properties (Department of Environment, South Africa 2000). These phenomena indirectly change the climatic processes that exert a strong influence on dry lands vegetation, biomass and diversity (Joo-Heon et al. 2016). High temperatures and low precipitations result because of high climate variability, leading to poor organic matter production and oxidation that make dry lands susceptible to wind and water erosions (World Meteorological Organization [WMO] 2005). Extreme weather conditions such as high temperatures and low precipitation constitute some of the causes of land degradation, which also cause drought (Laghrour, Moussadek & Mrabet 2016; World Health Organization [WHO] 2015). It is therefore possible where drought causes land degradation. Literature indicates that these two phenomena are positively correlated and the latter is a huge problem in Africa with over 90% of the land classified as arid, semi-arid and sub-humid (Environmental Monitoring Group 2015).

Lesotho has recently become a statistic among other southern African countries that are ravaged by severe drought to an extent that the Lesotho Government declared a state of drought emergency in the country on 22 December 2015. This drought disaster has compelled the country to appeal for assistance from the international community (WFP 2015a). This declaration came as result of the country being incapable of addressing water, food, nutritional support and medication to vulnerable people and to prevent further loss of livestock with a shortfall of $27.4 million. This money is intended to assist not only the most vulnerable groups but also people in the urban areas as well to meet high cost of food. Table 1 shows Lesotho and other southern African countries that are affected by the current El Niño situation which has caused drought events (South African Government 2012). This study was probed by a study in the Free State province of South Africa, which neighbours Mafeteng district of Lesotho in which the entire region was found to be 100% spatially covered with severe drought in 2015, more especially in the northern (Fezile Dabi district) and eastern (Thabo Mofutsanyana district) parts of the province (Hlalele 2015; Hlalele, Sakulski & Jordaan 2015). Given the high livelihood dependency on rain-fed agriculture and vulnerability to drought impacts in Lesotho and mountainous topography, the study aimed at estimating land degradation vulnerability from drought indices for effective mitigation and disaster risk reduction measures.

**TABLE 1:** Southern African countries’ rural populations affected by 2015 drought.

Country	Rural population	Affected population	Rural population (%)
Angola	12 767 654	800 000	6.3
Botswana	875 106	30 318	3.5
DR Congo	40 970 888	6 591 535	16.1
Lesotho	1 541 072	463 936	30.1
Madagascar	15 727 662	459 319	2.9
Malawi	14 492 248	2 833 212	19.5
Mozambique	18 384 814	137 784	0.7
Namibia	1 276 090	370 316	29.0
South Africa	18 828 580	14 069 662	74.7
Swaziland	1 011 606	200 897	19.9
Tanzania	36 762 641	424 136	1.2
Mauritius	180 981 810	28 670 087	15.8
Zambia	9 168 601	798 948	8.7
Zimbabwe	10 174 849	1 490 024	14.6

*Source*: WFP, 2015b, *Food security analysis*, viewed 22 January 2016, from http://vam.wfp.org/CountryPage_assessments.aspx?iso3=LS

## Study area

Lesotho is a small, lower-middle-income country in southern Africa that is fully engulfed in South Africa with an estimated area of 30 000 km^2^ (WFP 2013). This country is divided into 10 administrative districts, one of which is Mafeteng that falls among the most vulnerable to climate change variability (WFP 2013). Mafeteng district in particular comprises lowlands and a small portion of foothills whose soils are most susceptible to both wind and soil erosions because of poor rangelands management (Bureau of Statistics [BOS] 2010:1). In a recent study undertaken by Belle and Hlalele (2015) on assessment of agricultural drought vulnerability in Koti-Se-Phola Community Council, Mafeteng, this district proved to have a high level of vulnerability emanating from social and environmental sources.

## Literature review

The review of literature firstly provides a basic understanding of the key terms used in this research and finally an overview of the causes and impacts of land degradation.

### Definitions of terms

According to the International Federation of Red Cross and Red Crescent Societies (IFRC 2015), *vulnerability* is defined as diminished capacity of an individual, group or a system to anticipate, cope, resist or even to recover from an impact of either a natural or man-made hazard. In this study, vulnerability to land degradation is assessed in the drought hit Free State province of South Africa. *Land degradation* is a process that leads to a significant reduction of productive capacity of a land (Skidmore 1986; United Nations Environment Programme [UNEP] 2015). *Hazard* refers to a dangerous condition, substance or a phenomenon that may lead to loss of life, health impacts, injury, property damage, loss of livelihood, socio-economic disruption as well as environmental damage (United Nations International Strategy for Disaster Reduction [UNISDR] 2009). Therefore, from the above definitions, land degradation is previewed as a hazard that disrupts the livelihood of South Africans because Free State is said to be one of the main staple food (maize) producers in the country. *Disaster* is defined as a serious disruption of the functioning of a system or community resulting in widespread of adverse effects that undermine the available resources of that particular system (UNISDR 2009). Wind erosion climatic erosivity is a measure of the climatic tendency to provide suitable conditions for wind erosion. Wind erosion occurs when the shear force exerted on the surface exceeds that which binds the surface materials together; therefore, strong winds and dryness increase soil susceptibility to erosion (Skidmore 1986).

### Causes and impacts of land degradation

Land degradation has reduction effects on crop and pasture productivity, soil fertility, salinity and ground cover as well on fuel wood, which are directly linked with poverty and food insecurity (UNEP 1987). Land degradation mostly affects the agricultural industry with the following impacts: deterioration in the chemical and physical properties of soils, acceleration of soil loss, reduction in primary productivity of plant communities, decline in biodiversity and increase in hazards for human occupancy (Department of Natural Resources 1999). Other causes of this phenomenon include deforestation, overgrazing, poor land management practices, fires and drought events leading to increased poverty levels and food insecurity (Birdlife South Africa 2010; Low 2013; Mondal 2015; World Wildlife Fund [WWF] 2015). Similarly, UNCCD (2015) asserts that drought aggravates land degradation and climate change on the other hand aggravates the former.

## Materials and methods

*Standardised precipitation index* (SPI) is simply defined as a normalised index that represents a probability of a rainfall occurrence of an observed rainfall amount compared to the rainfall climatology at a certain geographical location over a long-term reference period (Man-Chi 2013). Moreover, National Center for Atmospheric Research (NCAR 2015) asserts that SPI is a probability index that provides a better representation of both abnormal wetness and dryness than any Palmer indices such as Palmer Drought Severity Index (PDSI). The merit of this index is that it can be computed for various time scales, hence providing early warning of drought and its severity (Jha 2010). This index is well suited for risk management purposes (Hayes 2006). Another advantage of this index is that precipitation is the only input parameter in its computation making it less complex. However, the weakness of this index is that it can only quantify the precipitation deficit, values based on preliminary data may change, and values change as the period of record grows (Belayneh & Adamowski 2012:2; WMO 2012:4). Table 2 shows values of SPI.

**TABLE 2:** Standardised precipitation index values.

SPI value	Category	Probability %
> 2.0	Extremely wet	2.3
1.5 to 1.99	Very wet	4.4
1 to 1.49	Moderately wet	9.2
−0.99 to 0.99	Near normal	34.1
−1.0 to −1.49	Moderately dry	9.2
−1.5 to −1.99	Severely dry	4.4
< −2.0	Extremely dry	2.3

*Source*: Tsakiris, G., Loukas, A., Pangalou, D., Vangelis, H., Tigkas, D., Rossi, G. et al., 2015, *Drought characterization*, pp. 85–102, School of Rural and Surveying Engineering, National Technical University of Greece, National Technical University of Greece, Greece and World Meteorological Organization (WMO), 2012, *Standardized precipitation index user guide*, United Nations, Geneva, Switzerland, p. 4

SPI, standardised precipitation index.

The SPI calculations are based on accumulation of precipitation for fixed time scale of interest (e.g. 3, 6, 9, 12, … months), then such as series is fitted into a gamma probability distribution which is suitable for this climatological precipitation time series (Tsakiris et al. 2015). The gamma distribution is defined by the following density function:

$$g(x)=\frac{1}{\beta^{\alpha }\Gamma (\alpha )}x^{\alpha -1}e^{-x/\beta }\;\text{for}\;\text{x}>0$$ [Eqn 1]

Where α (shape parameters) and β (scale parameter) are estimated for each station as well as for each month of the year:

$$\begin{array}{l} \alpha =\frac{1}{4A}\left(+\sqrt{1+\frac{4A}{3}}\right)\\ \beta =\frac{\bar{\text{x}}}{\alpha } \end{array}$$ [Eqn 2]

where $\text{A}=\ln(\bar{\text{x}})-\frac{\sum \ln(x)}{n}$, and *n* = number of obeservations.

After these parameters have been estimated, their resulting values are used to calculate cumulative probability as follows:

$$G(x)=\int_{0}^{x}g(x)dx=\frac{1}{\beta^{\alpha }\Gamma (\alpha )}\int_{0}^{x}x^{a-1}e^{-x/\beta }dx$$ [Eqn 3]

In cases where *t* = x/β, an incomplete gamma function becomes:

$$G(x)=\frac{1}{\Gamma (\alpha )}\int_{0}^{x}t^{\alpha -1}e^{-1}dt$$ [Eqn 4]

As the gamma function is undefined at x = 0, the cumulative probability is calculated from the following equation (Tsakiris et al. 2015):

H(x)=q+(1−g)G(x) [Eqn 5]

Where *q* is the probability of a zero and *G*(*x*) is the cumulative probability of the incomplete gamma function. If *m* is the number of zeros in a precipitation time series, then *q* can be estimated by *m/n*. The cumulative probability is then transformed to the standard normal random variable *z* with mean zero and variance one, which is the value of the SPI (Tsakiris et al. 2015). Precipitation data were provided by the Lesotho Meteorological Services. This monthly precipitation data had gaps which were filled by average values for computation. A Drought Indices Calculator (DrinC) developed by Tsakiris and Vangelis (2005) in Greece was used to compute SPI values in gamma function. The results were presented in tables and graphs. Prior to the analysis, a Pettitt’s test for homogeneity was conducted to avoid spurious results because of fluctuations in the mean and variance.

The collected data were analysed in two dimensions: severity and frequency from (SPI-3 and SPI-annual time step).

Severity was calculated from the following equation:

$$S_{e}={\left|\sum_{j=1}^{m}Index_{j}\right|}_{e}$$ [Eqn 6]

Where S_e_ is the absolute value of all indices in the range (< −1) of ‘m’ months/seasons/years. According to Table 2, drought starts when SPI values fall below −1; hence, this was selected as the threshold value. Drought frequency (F_s_) was used to analyse the drought liability during the study period:

$$F_{s}=\frac{n_{s}}{N_{s}}\times 100$$ [Eqn 7]

Where *n*_*s*_ and *N*_*s*_ denote the number of months whose indices fall in the interval (< −1) and the total months/seasons/years during the study, respectively. The current study used monthly time scale.

## Results presentation and discussion

**FIGURE 1:**
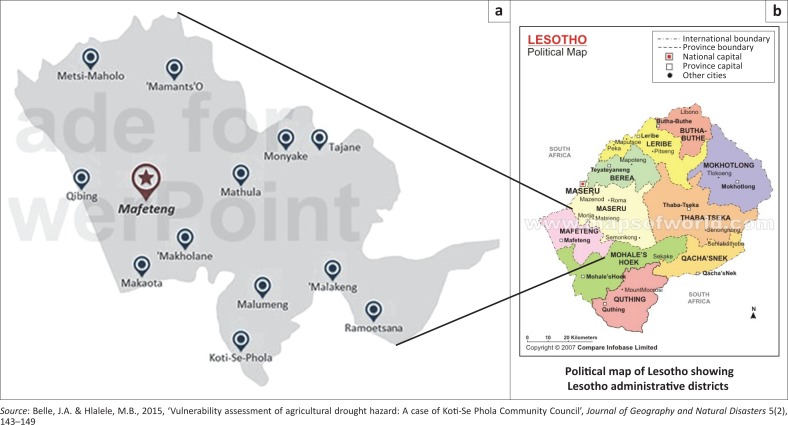
Study area, Mafeteng district in Lesotho. (a) Mafeteng district and (b) Lesotho.

**FIGURE 2:**
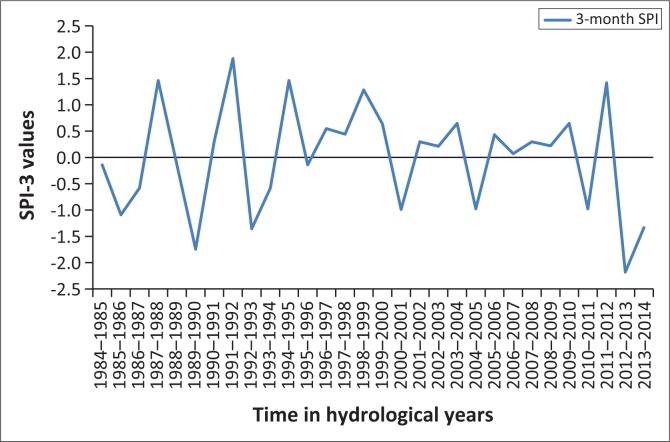
Three-month standardised precipitation index: January–March, 1984–2014.

**FIGURE 3:**
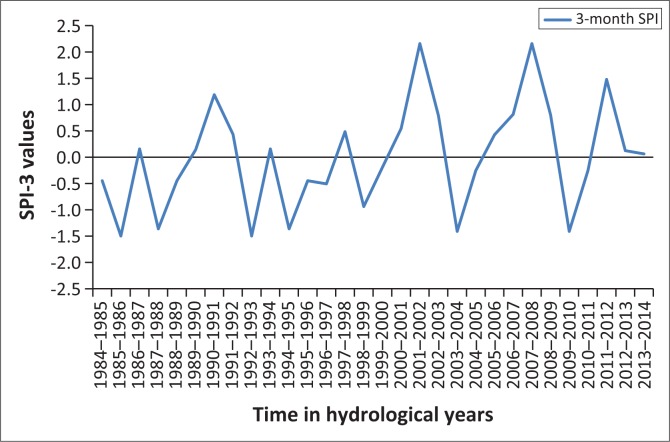
Three-month standardised precipitation index: April–June, 1984–2014.

**FIGURE 4:**
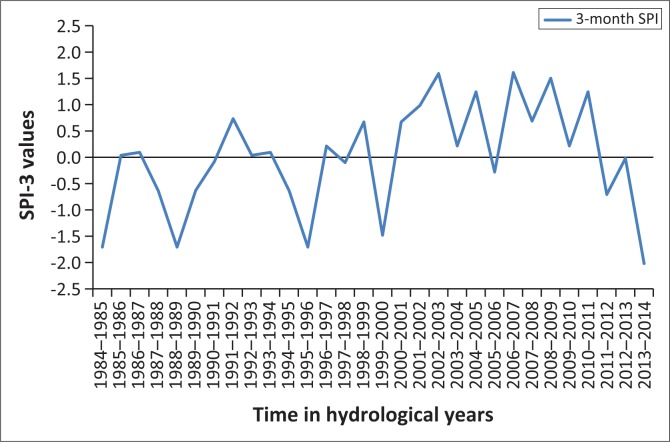
Three-month standardised precipitation index: July–September, 1984–2014.

**FIGURE 5:**
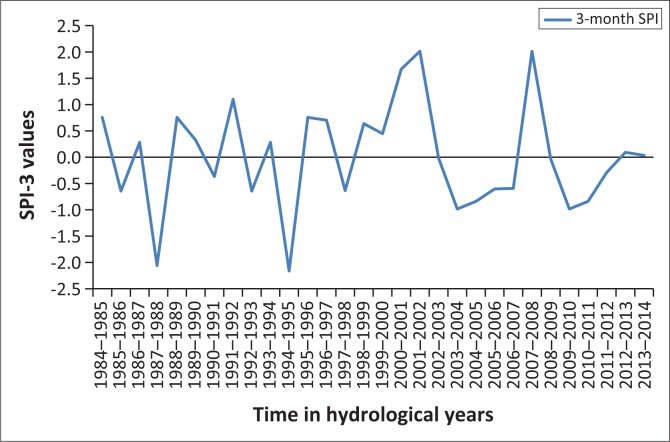
Three-month standardised precipitation index: October–December, 1984–2014.

Figures 1–5 show the 3-month SPI plots from 1984 to 2014 on different seasons and annual basis respectively. Table 3 shows the results of a non-parametric Pettitt’s test. The results show that the precipitation data were homogenous with a 2-tailed *p*-value = 0.320 > 0.05. The seasonal plots are followed by detailed analysis in Table 4.

**TABLE 3:** Non-parametric homogeneity test (Pettitt’s test).

Pettitt’s test (26,3)	Parameters
*K*	5688.000
*t*	2002.000
*p*-value (two-tailed)	0.320
alpha	0.050

**TABLE 4:** Drought analysis in terms of frequency and severity over the 1984–2014 period with standardised precipitation index < −1.

Time step	Drought years	Drought frequency %	Severity
Jan.–Mar.	1985–1986, 1989–1990, 1992–1993, 2012–2013, 2013–2014	5/30 = 17%	7.71
Apr.–June	1985–1986, 1987–1988, 1992–1993, 1994–1995, 2003–2004, 2009–2010	6/30 = 20%	8.55
July–Sept.	1984–1985, 1988–1989, 1995–1996, 1999–2000, 2013–2014	5/30 = 17%	8.62
Oct.–Dec.	1987–1988, 1994–1995	2/30 = 7%	4.32
Annual	1985–1986, 1992–1993, 2012–2013, 2013–2014	4/30 = 13%	5.72

**FIGURE 6:**
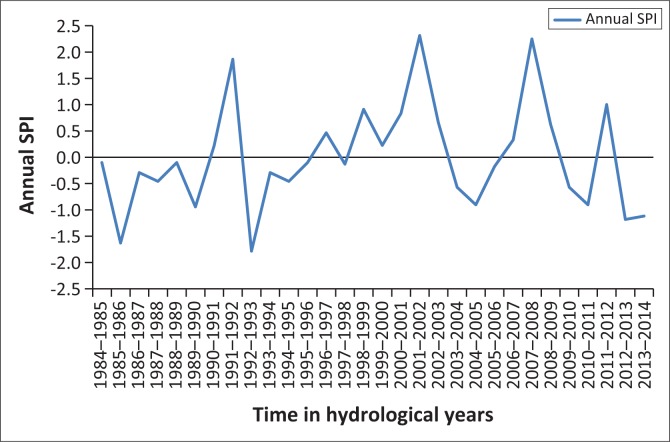
Annual standardised precipitation index: 1984–2014.

From Table 4, it can be deduced that April–June term season was the most frequent and severe with droughtiest year with all year dry years approximately the same. However, on the contrary, October–December seems to be the least dry season. The periods 2012–2013 and 2013–2014 have been the severely dry seasons of all years over the entire study period. This confirms the slow onset nature of drought hazard, whose presence was seen in December 2015 by the Lesotho Government. Similarly, these 2 years are revealed by the January–March, July–September and annual SPI values giving a clear picture of the current 2015 drought disaster in Lesotho. The drought frequency proved to be uniform over the study period years, with an accelerated severity in 2012–2013 and 2013–2014 years as seen earlier. Most dry periods were detected by the seasonal time scales. Figure 6 shows how drought frequency and drought severity correlate. The Pearson correlation coefficient is computed to be *r* = 0.96, showing a strong relationship of the two variables.

**FIGURE 7:**
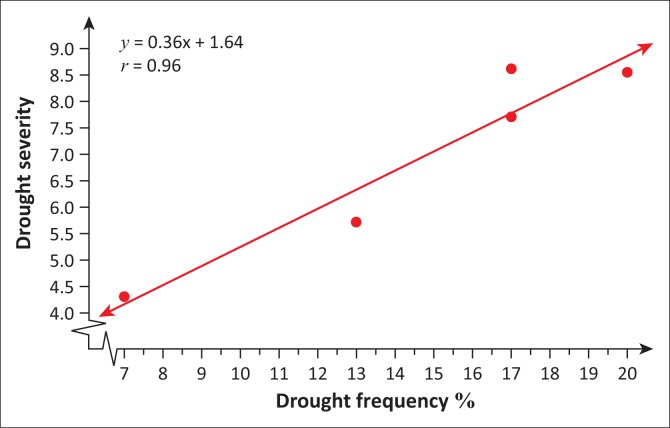
Scatter plot of drought severity against drought frequency.

A further analysis was made over decades to characterise drought in Mafeteng district. Table 5 depicts hazard analysis in three decades, which reveal that drought episodes in the study area return every 3 years approximately. Figure 7 shows how drought severity trends with frequency.

**TABLE 5:** A decal drought frequency (1984–2014).

Decades	Drought frequency (SPI −1)
1984–1993	2
1994–2003	2
2004–2014	1
Average	2.5 = 3 (rounded off)

SPI, standardised precipitation index.

## Conclusion

Given the land degradation percentage loss over the entire African continent, and a strong correlation between land degradation and both soil and wind erosion, it is clear that this situation is set to continue with high chances to increase. The vulnerability to land degradation seems to be constant over the years. Given high rain-fed agriculture livelihood dependency of over 80% Basotho population and mountainous Lesotho topography, it is imperative that the government sets up both environmental and livelihood community projects to prevent further adverse impacts of land degradation on people’s livelihoods. In conclusion, vulnerability to land degradation in the study area seems to be a constant phenomenon over the years with a possibility of an increase given the vast changing climate variability.
